# An Evaluation of the Cobas4800 HPV Test on Cervico-Vaginal Specimens in Liquid versus Solid Transport Media

**DOI:** 10.1371/journal.pone.0148168

**Published:** 2016-02-01

**Authors:** Hongxue Luo, Hui Du, Kathryn Maurer, Jerome L. Belinson, Guixiang Wang, Zhihong Liu, Lijie Zhang, Yanqiu Zhou, Chun Wang, Jinlong Tang, Xinfeng Qu, Ruifang Wu

**Affiliations:** 1 Department of obstetrics/gynaecology, Peking University Shenzhen Hospital, Shenzhen, PR China; 2 Shenzhen Key Laboratory on Technology for Early Diagnosis of Major Gynecological diseases, Shenzhen, PR China; 3 Gynecologic Oncology Division, Women’s Health Institute, Cleveland Clinic, Cleveland, Ohio, United States of America; 4 Preventive Oncology International, Cleveland Heights, Ohio, United States of America; State University of Maringá/Universidade Estadual de Maringá, BRAZIL

## Abstract

**Objectives:**

Determine the ability of the Cobas 4800 assay to detect high-risk human papillomavirus (HrHPV) and high-grade cervical lesions when using cervico-vaginal samples applied to liquid medium and solid media cards compared to a direct cervical sample.

**Methods:**

Two cervico-vaginal specimens (pseudo self-collected) were obtained from 319 women. One was applied to an iFTA Card (FTA) then the brush placed in liquid-based medium (LSELF); the other was applied to a new solid media: POI card (POI). The clinical performance of Cobas4800 assay using the three aforementioned specimens was compared to direct collected endocervical specimens in liquid media (LDOC).

**Results:**

The overall agreements of HrHPV detection were 84.2% (LSELF vs. LDOC), 81.0% (FTA vs. LDOC), and 82.3% (POI vs. LDOC). LSELF, FTA and POI identified 98.0%, 79.6%, and 97.5% positive cases of LDOC. Sensitivity to identify CIN2+ were 98.4% (LSELF), 73.8% (FTA), 95.1% (POI), and 93.4% (LDOC) respectively. FTA had 78.1% and 90.4% agreement with the LSELF samples for all HrHPV and HPV16/18 detection respectively, while POI had 91.6% for both.

**Conclusions:**

Cobas4800 HPV test combined with cervico-vaginal specimens applied to both liquid media and POI solid card are accurate to detect HrHPV infection and high-grade cervical lesions as compared with direct endocervical samples in liquid media.

## Introduction

Cervical cancer is the fourth most common female tumor world-wide, with an estimated 500,000 new cases annually, causing over 270,000 deaths in 2012 [1–3]. It is universally acknowledged that almost all cervical cancers and immediate precancerous lesions are caused by persistent infection with at least one of 15 high-risk HPV genotypes (HrHPV). HPV16 and HPV18 are the most carcinogenic of these types and are responsible for approximately 70% of all cervical cancers throughout the world [4–7].

To increase the coverage for population-based screening programs world-wide, self-collected cervico-vaginal specimens provide a potential solution. Their equivalent sensitivity to physician collected samples has been demonstrated when combined with PCR based HrHPV testing methods [8–11].

In April 2014 the US FDA approved the first HPV assay for primary screening [12]. This assay, the Roche Cobas HPV test (Roche Inc., Pleasanton, CA, USA) is a qualitative multiplex assay, offering concurrent partial genotyping for types16 and 18 and detecting 12 other HrHPV genotypes as a pooled result [13]. The assay was first approved in the US for cervical cancer screening in 2011 [14].

A key component for implementation of self-collection programs is specimen transport. Recently, an estabished solid media transport card (iFTA,Whatman/GE Healthcare,United Kingdom)was introduced as an attractive preservative and transport medium for HPV DNA. With a non-liquid transport media, self-collection could potentially be simplified for the patient. The small format and considerable stability at room temperatures makes the card particularly suitable for transport from rural health care centers to the laboratory. In addition the card has the advantage of being amenable to molecular detection of HPV with simple DNA elution steps, requiring only a water-heat reaction. The concordance of the iFTA card in comparison with conventional liquid-based media for the detection and genotyping of HrHPV as well as diagnostic accuracy for high-grade cervical lesions has been reported [15–17]. Recently a new investigational card, the Preventative Oncology International (POI) card has been developed by Hyde Biomedical Corp (Wuhu City, Anhui, PR China) and evaluated. Reliable HrHPV detection and color stability in humid environments were reported [18].

Our study was designed to address the above self-collection issues. How effective is the Cobas4800 assay in detecting HrHPV and CIN2/3+ from self-collected samples? What is the relative performance of self-collected samples transported in liquid, or the iFTA, and POI cards compared to clinician-collected cervical specimens?

## Materials and Methods

### Study Subjects and sample collection

Women referred to the Peking University Shenzhen Hospital Center for Cervical Diagnosis or the Shenzhen Pingshan Maternal and Child Health Hospital in China between January 19^th^ and 28^th^, 2015 were eligible for the study. The reasons for referral were abnormal cervical visual inspection, positive testing for high-risk HPV or abnormal cytology. Pregnant patients were excluded.

“The Development and Evaluation of a New Solid Media Specimen Transport Card for Population Based Cervical Cancer Prevention” was approved by both the Cleveland Clinic (Cleveland, Ohio) and Peking University Shenzhen Hospital IRBs. This study is a side-study of that project.

In the conduct of the study examinations, 2 simulated self-collections (**FTA** and **POI**) were obtained by a physician (cervico-vaginal specimens without using a speculum), followed by placement of a vaginal speculum to obtain a direct endocervical specimen (**LDOC**).

The“pseudo self-collections”were done in alternating order based on study ID number. In brief, two nylon conical cervical sampler brushes (Just For Me^®^ brush, CE-marked, Preventive Oncology International, Inc. Cleveland Heights, Ohio)were sequentially inserted into the upper vagina and rotated 3 times. Subsequently, the brushes were rolled across the paper contained in either the FTA cartridge (FTA), or the new POI card and air dried [18]. The brush used for the iFTA card was cut from the shaft and dropped into a collection vial containing 3 mL SurePath liquid.(SurePath;Tripath Imaging,Inc.,Burlington,NC27215,USA)(**LSELF**) [19,20]. Therefore LSELF represents a liquid split-sample from the iFTA card.

**Fig 1:**
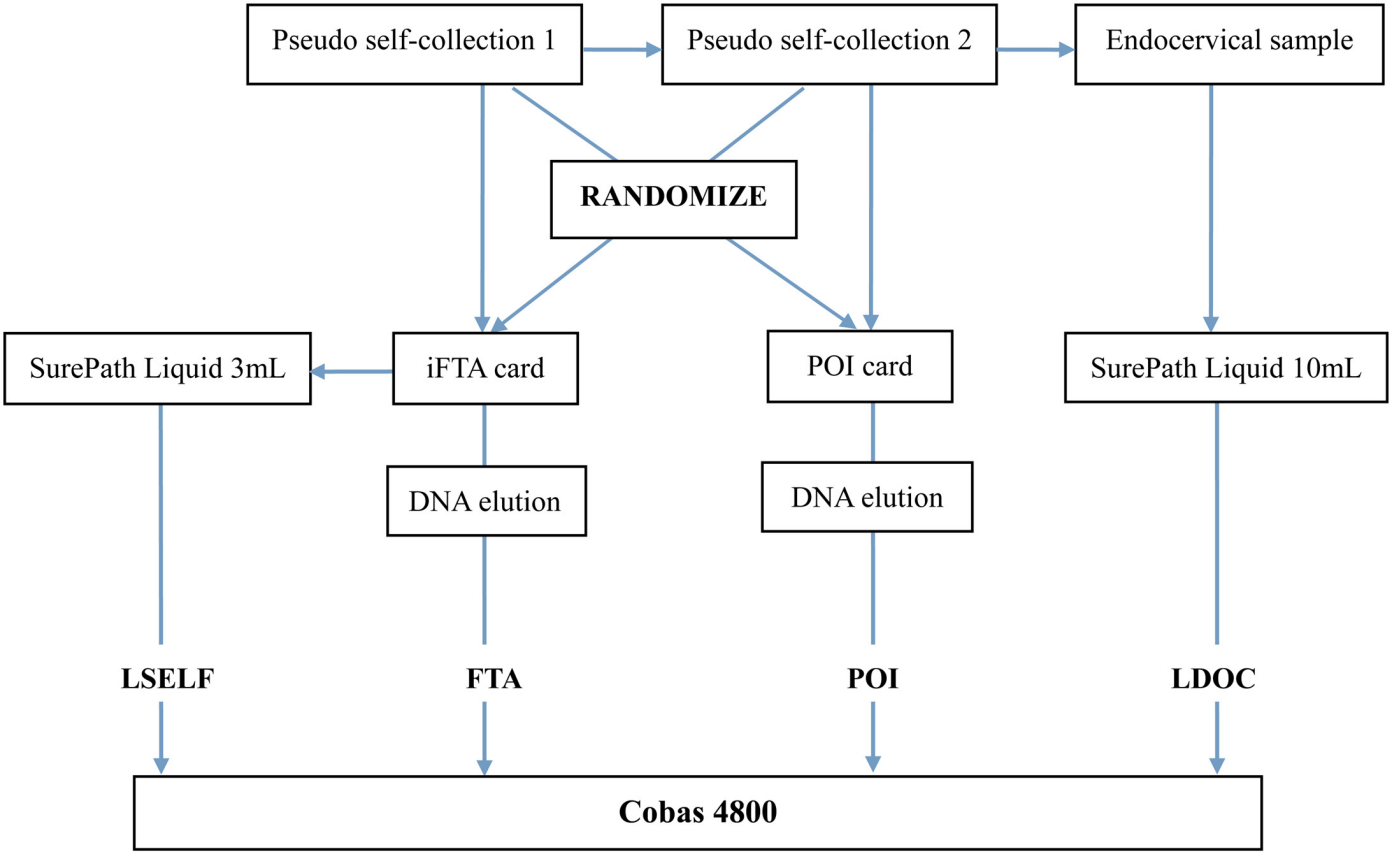
Flow diagram.

A speculum was then inserted into the vagina and a direct endocervical sample obtained using a Rovers Cervex-Brush (Rovers Medical Devices B.V.,Oss, The Netherlands). The brush head was then suspended in a 10 mL vial of SurePath Preservative Fluid (LDOC). All liquid vials were stored at a temperature of 4°C awaiting further processing together with the solid cards. All samples were tested by Cobas4800 within two weeks of collection (Fig 1).

After obtaining the study specimens, all women either underwent a colposcopy examination using the POI micro-biopsy protocol of directed and random biopsies [21], or a pre-scheduled LEEP. Pathology specimens were interpreted by a cervical pathology specialist at Peking University Shenzhen Hospital (au CW).

### HPV Detection/genotyping by Cobas4800 assay

The Cobas4800 HPV Test was Conformité Européenne (European Community) CE-markedin 2009 and received US Food and Drug Administration approval in April 2011[22]. The cobas 4800 system platform (Roche Molecular Diagnostics,Pleasanton,CA.), consists of the cobas x480 instrument and the cobas z480 analyzer. It features fully automated nucleic acid extraction in combination with real-time PCR technology plus software that integrates the two components. HPV 16 and 18 are identified separately while the other 12 HR-HPV types (31,33,35,39,45,51,52,56,58,59,66 and 68) are detected as a pool. The human cellular gene beta-globin is used as an internal control to measure the sample adequacy as well as the quality of extraction and amplification.

We used two different specimen preparation procedures for the cobas z480 that had previously been optimized. Nucleic acids used for the Cobas assay from the two liquid specimens(LDOC and LSELF), were prepared using cobas x480. The instrument could yield 50 μL of nucleic acid, eluted from 500 μL of SurePath solution used per subject. The eluted DNA solutions from the cards (FTA and POI)were prepared according to the Cobas4800 device “special instructions”. The“PCR ONLY”program was performed using cobas z480. 5μL DNA solution from the POI card sample and 10μL DNA solution from the iFTA card sample were needed to ensure a sufficient sample (adequate DNA)was present for valid detection. All samples(**LDOC, LSELF, FTA,** and **POI**)from the same patient were tested using the cobas z480 assay following the manufacturer’s instructions.

### Retrieval of DNA from the iFTA and POI cards

The iFTA card, based on Whatman 903^®^ paper, is impregnated with patented chemical reagents that lyse cells upon contact, immobilizing and stabilizing nucleic acids from freshly applied samples. Additionally, the paper contains an indicating dye that changes color from purple to yellow when a clear sample is applied showing the location of the samples proteins [23]. The yellow on the FTA cartridges was punched using a 3-mm Harris micro-punch (BSD, USA). Three punches were obtained from every sample card and they were all placed in a single well in a 96-well plate. They were then all washed once using 100 microliters sterile water. The water was then carefully removed with a sterile fine-tip pipette. The DNA elution was performed in 50 microliters of sterile water at 56°C for 30 min immediately followed by 95°C for 15 min. in a heating block. The 96-well plate containing DNA elution and pieces of card were centrifuged at 4000 rpm for 3 min and the eluted DNA was transferred into a new 96-well plate being stored at -80°C for further use.

The Preventative Oncology International (POI) card consists of PK 226^®^ paper (PerkinElmer, Greenville, SC) treated with a combination of a lysing solution and a dye (Hyde Biomedical Corporation, Wuhu, Anhui, China). The lysing solution contains an ionic detergent to lyse cell membranes and stabilize DNA as well. Similar to the iFTA card, the indicating dye changes color when the sample is applied (blue to pink). DNA retrieval followed a protocol similar to the iFTA card.

### Statistical analysis

Agreement between samples was measured by absolute agreement and Kappa statistics (Cohen’s Kappa). Fisher’s exact test and McNemar’s Chi-square were performed to calculate differences between paired proportions at a probability level of 0.05. All data were analyzed using SAS version 9.2 (SAS Inc., Cary, NC).

Hyde Biomedical Corporation, the designer of the chemistry for the PK paper and Preventive Oncology International, the designer of the POI card, had no involvement in the clinic or laboratory and were blinded to all laboratory testing until completed. GE Healthcare likewise had no involvement in clinic or laboratory testing.

## Results

### Participants

319 eligible women between 19 and 71 years of age signed an informed consent document agreeing to participate after being educated on the aims and specific conduct of the study. We excluded eight women with at least one of their specimens with inadequate cellularity or DNA content (2 LDOC, 3 FTA, and 3 POI card samples). Therefore, the final analytic cohort was restricted to 311 women (97.5%) with complete HPV data. The median age was 39 years (SD 10.8 years). 18/311 patients were arriving for treatment of prior biopsy results and did not have biopsies done at their study clinic visit. 17/18 received cryotherapy for CIN 1/persistent HPV as is common in China, with 1/18 not being treated. Therefore 293 patients are available for analysis of disease endpoints.

### HrHPV Detection

The HPV positivity of LDOC, LSELF, FTA and POI specimens were 64.6%(201/311), 77.8%(242/311), 57.2%(178/311) and 79.1%(246/311) respectively.

We compared the agreement of HrHPV detection between LDOC specimens and the other three specimens, as shown in Table 1. The overall agreements were 84.2%(LDOC vs LSELF), 81.0%(LDOC vs FTA), 82.3%(LDOC vs POI) respectively. Restricting the HrHPV detection to the CIN2+ subset, SELF and POI card specimens had a very good overall agreement (95.1% and 91.8% respectively) and positive agreement (95.0% and 91.7% respectively) with the LDOC specimens. In the same subset the FTA specimens demonstrated lower agreement (79.0%). If the HrHPV detection result of Cobas4800 using LDOC specimens is used as the golden standard, the sensitivity of SELF and POI specimens for HrHPV detection reached 98.1% and 97.5% respectively, and the FTA sensitivity was 79.6%.

**Table 1:** Agreement of HrHPV detection between cervical and cervico-vaginal specimens applied to both liquid media and solid card in different subsets by Cobas4800 HPV Assay^a^.

	LDOC hrHPV+	LDOC hrHPV-	% Positive	% Overall	Kappa Value	P-values^b^
	(n = 201)	(n = 110)	Agreement	Agreement	[95% CI]	
**LSELF**						
+	197	45	80.08%	84.24%	0.624	0.25
-	4	65			[0.532–0.715]	
**FTA**						
+	160	18	73.06%	81.03%	0.604	0.004
-	41	92			[0.514–0.693]	
**POI**						
+	196	50	78.09%	82.32%	0.574	<0.001
-	5	60			[0.479–0.669]	

^**a**^,HrHPV+, positive for high-risk HPV; HrHPV−, negative for high-risk HPV; 95% CI, 95% confidence interval; LDOC, cervical samples in liquid media;LSELF, cervicovaginal specimen in liquid media; FTA, cervicovaginal specimens applied to iFTA card; POI, cervicovaginal specimens applied to POI card;

^***b***^, Calculated using the exact McNemar’s Chi-square test.

### Disease Endpoints

Among the 293 cases with biopsy results, 39.3%(115/293) demonstrated normal histology. The prevalence of CIN2+ and CIN3+ were 20.8%(61/293) and 11.9%(35/293) respectively. The sensitivity of four different specimens for detecting CIN2+ were 93.4% for LDOC, 98.4% for LSELF, 73.8% for FTA, and 95.1% for POI. For CIN3+, both the LDOC and LSELF specimens had a sensitivity of 100%, while the FTA was 77.1% and POI 97.1%.Table 2

**Table 2:** Clinical performance of the Cobas4800 HPV Assay for CIN2+ and CIN3+ in different samples^a^.

	CIN2+ (n = 61)			CIN3+ (n = 35)		
	Sensitivity	P-values^b^	Specificity	Sensitivity	P-values^b^	Specificity
	[95% CI]		[95% CI]	[95% CI]		[95% CI]
**LDOC**	93.44%		42.67%	100.00%		39.92%
	84.05%-98.18%		36.22%-49.31%	90.00%-100.00%		33.90%-46.18%
**LSELF**	98.36%		20.02%	100.00%		25.58%
	91.20%-99.96%	1	22.34%-34.27%	90.00%-100.00%	1	20.37%-31.36%
**FTA**	73.77%		46.12%	77.14%		44.57%
	60.93%-84.20%	0.006	39.58%-52.76%	59.86%-89.58%	0.005	38.41%-50.87%
**POI**	95.08%		25.43%	97.14%		23.64%
	86.29%-98.97%	1	19.96%-31.54%	85.08%-99.93%	1	18.59%-29.31%

^**a**^, LDOC, cervical samples in liquid media; LSELF, cervicovaginal specimen in liquid media; FTA, cervicovaginal specimens applied to iFTA card; POI, cervicovaginal specimens applied to POI card;

^**b**^, Calculated by using the exact McNemar’s Chi-square test, compared to cervical samples in liquid media

There was no significant difference in sensitivity for both the detection of CIN2+ and CIN3+ between LDOC and LSELF (p = 1.00). The LDOC and POI specimens were also similar (p = 1.00). However LDOC was significantly more sensitive than FTA for both the detection of CIN2+ and CIN3+ (p = 0.006 and p = 0.005 respectively).

### Genotype Detection

The HrHPV results were categorized into 3 groups and analyzed according to genotype based on cancer risk: HPV16 and/or 18 positive, Non-16/18 positive, and HrHPV negative. Risk stratification between LDOC and the other three specimens detected by Cobas4800 were all in good agreement: Kappa = 0.719(LDOC/LSELF), 0.686(LDOC/FTA), 0.694(LDOC/POI) (Table 3). The detection of HPV16/18 using LDOC, LSELF, FTA, and POI by Cobas4800 were 19.6%(61/311), 23.2%(72/311), 15.4%(48/311), and 25.7%(80/311) respectively. Furthermore, we analyzed the agreements between LSELF and the solid card samples (FTA and POI) for three independent risk groups as well as just HrHPV positive. As shown in Table 4, the positive agreement between LSELF and POI card is over 91% with Kappa over 0.75. FTA agreement with LSELF for HPV16/18 types detection was lower, demonstrating good agreement (90.4%, kappa = 0.693)

**Table 3:** Comparison of HPV test results in cervical samples collected in liquid media and cervico-vaginal specimens applied to both liquid media and solid cards categorized by HPV cancer risk^a^.

	LDOC			Kappa
	HPV16/18	Non-16/18	HrHPV Neg	[95% CI]
**LSELF**				
HPV16/18	57	7	8	
Non-16/18	2	131	37	
HrHPV Neg	2	2	65	
				0.719
				0.635–0.802
**FTA**				
HPV16/18	46	2	0	
Non-16/18	2	110	18	
HrHPV Neg	13	28	92	
				0.686
				0.597–0.776
**POI**				
HPV16/18	58	15	7	
Non-16/18	1	122	43	
HrHPV Neg	2	3	60	
				0.694
				0.615–0.774

^**a**^, HPV16/18 (HPV16 and/or 18 positive); Non-16/18 (Non-16/18 positive); LDOC, cervical samples in liquid media; LSELF, cervicovaginal specimen in liquid media; FTA, cervicovaginal specimens applied to iFTA card; POI card, cervicovaginal specimens applied to POI card;

**Table 4:** Agreement of Stratifying detection for different HPV risk groups between cervico-vaginal specimens applied to liquid media and solid cards^a^.

	LSELF vs FTA				LSELF vs POI			
	%Agreement	Kappa[95% CI]		P-values^b^	%Agreement	Kappa[95% CI]		P-values^b^
**all HrHPV**	78.14%	0.524	[0.435–0.614]	<0.001	91.64%	0.753	[0.663–0.843]	0.557
**HPV16/18**	90.35%	0.693	[0.593–0.794]	<0.001	91.64%	0.774	[0.691–0.856]	0.169
**Non-16/18**	78.14%	0.658	[0.578–0.738]	<0.001	91.64%	0.767	[0.695–0.839]	0.618
**HrHPV neg**	82.64%	0.524	[0.435–0.614]	<0.001	88.42%	0.753	[0.663–0.843]	0.557

^**a**^, all HPV (any HPV positive); HPV16/18 (HPV16 and/or 18 positive); Non-16/18 (Non-16/18 positive); LSELF, cervicovaginal specimen in liquid media; FTA, cervicovaginal specimens applied to iFTA card; POI, cervicovaginal specimens applied to POI card.

^**b**^, Calculated by using the exact McNemar’s Chi-square test;

## Discussion

The introduction of HPV DNA testing is progressively changing conventional cervical cancer screening based on the Papanicolaou (Pap) cytology test, which has been used since the mid-1900s. Compared with Pap testing, HPV testing has consistently demonstrated superior sensitivity for the detection of high-grade precursor lesions (CIN2+) but lower specificity using a variety of assays. Accordingly, diagnostic triages are needed. Currently, less than 20 HPV genotypes are recognized to cause virtually all cervical carcinomas [6,24]. Types HPV16 and HPV18, associated with over 70% of all cervical cancers, provide a key triage option to address the prevalence of non-neoplastic HPV infection in the majority of women testing HrHPV positive[4,25,26].

The Cobas 4800 HPV test is the only approved test by US FDA that provides, in the primary screening assay, specific genotyping results for HPV types 16 and 18 as well as pooled results for 12 high-risk HPV types[27]. In North America and Europe, it has been clinically validated for ASCUS triage and in 2014 in the USA was approved for primary screening.

The success of cervical cancer screening programs in the western world has unfortunately not been realized by the world’s medically underserved where 85% of the cases of cervical cancer occur. Self-collection has been studied for many years as a way to reach these medically underserved populations as well as non-participants in organized screening programs [28,29]. Over the past several years self-collected samples transported in liquid media have repeatedly been shown to be as sensitive for the detection of high grade pre-cancer as a physician-obtained direct endocervical sample when paired with a PCR based assay [30–32]. Other studies have also discussed the feasibility of using iFTA cards as an alternative to conventional liquid-based media for clinician-collected cervical specimens [17,33], as well as self-collected cervico-vaginal specimens [16,34–36].

To our knowledge, all published trials of solid transport media for HPV detection have used the iFTA card. In this study we added the POI card, which has been recently validated to be able to lyse cell membranes and stabilize DNA reliably when contacting fresh samples similar to the iFTA card. The trial also demonstrated a superior ability of the POI card to transfer HrHPV and retain it’s normal color in high humidity environments compared to iFTA card [18].

This is the first study pairing the Cobas4800 HPV test with direct liquid samples (LDOC), as well as solid media cervico-vaginal specimens (FTA and POI), and liquid cervico-vaginal samples (LSELF). It is the first study of Cobas to assess the effective transfer of HrHPV for the detection of CIN2/3+ with a variety of transport options for self-collected samples.

In our study, both cervico-vaginal specimens (LSELF) and POI card samples could detect almost all (98.0% and 97.5%) HrHPV positive cases identified by Cobas4800 in direct cervical samples stored in liquid media (LDOC); whereas iFTA card samples detected 79.6%. The HrHPV positivity of iFTA card samples (FTA) were significantly lower than the other two samples (p<0.01). There were no significant differences between LDOC vs. LSELF or POI samples in detecting HrHPV or CIN2/3+ using Cobas4800. Compared with FTA, LSELF and POI specimens identified more HPV16/18 positive cases (23.2% and 25.7% respectively vs 15.4%; p<0.001) consistent with their higher sensitivity.

Our results comparing LDOC and LSELF for detecting both HrHPV and CIN2/3+ using Cobas4800 are similar to Stanczuk et.al. [37], whereas the overall agreement for all HrHPV positive cases between LDOC and LSELF in our study was lower (84.2% vs 94%).

The agreement of direct cervical samples and self-samples applied to iFTA cards in regards to HPV genotype has been observed previously [36]. However, in our trial to control the quality of collection, so as to create a purer evaluation of the technologies, we decided to use the “pseudo-self-sampling” model we have described.

In our study, the cervico-vaginal specimens were first applied to iFTA cards before being placed into a tube containing liquid-based medium (the LSELF specimen). A similar method has been used previously and the cervico-vaginal specimens showed a high level of agreement with cervico-vaginal specimens applied to iFTA cards for the detection of High-risk HPV [16]. However in our study, this agreement was only seen in regards to types 16/18. By contrast, the POI card demonstrated a strong agreement for HrHPV detection as well as after stratifying the HPV types according to risk.

For reasons that are unclear to us at this time, the Cobas4800 test in combination with iFTA card (FTA) was inferior to the other samples studied both for the detection of HrHPV as well as CIN2/3+. This was especially surprising with the comparison LSELF to FTA, since the LSELF sample was in fact a secondary sample (split-sample) from FTA (the primary sample). The influence of placing the brush head in only 3 mL of SurePath surely created a dense liquid sample. Again how this was seen differently by Cobas is unclear. The fact that 2x the amount of DNA (10μL for FTA and 5μL for POI) was needed for adequacy using Cobas suggests either the iFTA cards are less efficient at DNA transfer than the POI cards or there is some currently unrecognized interaction between the Cobas assay and the iFTA cards. It should be noted that all cards used were within their expiration dates.

Our next trial will explore true self-collection and direct collection, with liquids and cards combined with three distinct types of assays in a population based clinical trial. We feel the current study has clearly validated the use of the Cobas assay with self-collected samples transferred in both liquid and solid media cards. Options are important as we continue to pursue mass population screening using community based models, so our programs can be specific for the target populations we serve[38].
